# Initial Development of an Electronic Testis Rigidity Tester

**DOI:** 10.1100/tsw.2011.56

**Published:** 2011-03-22

**Authors:** Petros Mirilas, Odysseus Tsakiridis

**Affiliations:** ^1^ Centers for Surgical Anatomy and Technique, Emory University School of Medicine, Atlanta, GA, USA; ^2^ Department of Electronics, Technological Institute of Athens, Greece

**Keywords:** testis, rigidity, elasticity, tactile sensors, medical device design processes, cryptorchidism, torsion

## Abstract

We aimed to develop our previously presented mechanical device, the Testis Rigidity Tester (TRT), into an electronic system (Electronic Testis Rigidity Tester, ETRT) by applying tactile imaging, which has been used successfully with other solid organs. A measuring device, located at the front end of the ETRT incorporates a tactile sensor comprising an array of microsensors. By application of a predetermined deformation of 2 mm, increased pressure alters linearly the resistance of each microsensor, producing changes of voltage. These signals were amplified, filtered, and digitized, and then processed by an electronic collector system, which presented them as a color-filled contour plot of the area of the testis coming into contact with the sensor. Testis models of different rigidity served for initial evaluation of ETRT; their evacuated central spaces contained different, increasing glue masses. An independent method of rigidity measurement, using an electric weight scale and a micrometer, showed that the more the glue injected, the greater the force needed for a 2-mm deformation. In a preliminary test, a single sensor connected to a multimeter showed similar force measurement for the same deformation in these phantoms. For each of the testis models compressed in the same manner, the ETRT system offered a map of pressures, represented by a color scale within the contour plot of the contact area with the sensor. ETRT found certain differences in rigidity between models that had escaped detection by a blind observer. ETRT is easy to use and provides a color-coded “insight“ of the testis internal structure. After experimental testing, it could be valuable in intraoperative evaluation of testes, so that the surgeon can decide about orchectomy or orcheopexy.

## INTRODUCTION

Pathology may change the mechanical properties of tissues; thus, an estimation of elasticity (or in the opposite sense, stiffness or rigidity) may reflect the internal structure of the tissue. Palpation has been the

[*] Presented as a poster at the 4th International Greek Biotechnology Forum (IGBF4), Zappion Hall, Athens, Greece, February 2–3, 2008.

primary means of clinical examination to assess tissue mechanical properties. However, it is subjective, depending upon the examiner's experience. It also a qualitative method, which at best can be transformed into a semi-quantitative method by the use of a scoring system and, therefore, is not very useful in studies. To overcome this problem, research in the last 2 decades has focused on quantitative tissue assessment and disease diagnosis, and a separate branch, elasticity imaging, has emerged. Various modalities have been used to obtain measurements, e.g., ultrasound[1], MRI[2], or optical measurement[3], after mechanical disturbance of the tissue provoked by compression, indentation (by a rigid object, ultrasound, air or water jet, etc.), or suction vibration of acoustic stimuli[3].

Mechanical imaging is a branch of elasticity imaging that uses a probe with a pressure sensor array (therefore named "tactile imaging") that, like the human finger, compresses soft tissue and detects the resulting changes. This technique has been used successfully to assess the elasticity of organs such as the prostate, breast, and vaginal wall[4].

Here we adopt tactile imaging to assess the testis during surgery, with the aim of obtaining an immediate assessment of the internal structure. This crucial information may determine the choice of surgical treatment of cryptorchidism and torsion: fixation of the testis in the scrotum, if the testis is viable, or orchectomy, if the testis has severe histological lesions affecting its viability and future fertility[5,6]. Although biopsy is the "gold standard" for evaluating testicular lesions, there are concerns that it may further harm the testis[7,8]. Thus, the surgeon usually relies on palpation (evaluation of rigidity) or visual evaluation (evaluation of color and size).

In order to obtain an objective testis evaluation in cryptorchidism or torsion, we have previously developed a mechanical Testis Rigidity Tester (TRT), which measures the force (mNt) resisting a given deformation of the testis[9]. However, TRT usage has been quite difficult and time consuming, and results are expressed as a single value. Nonetheless, lesions are not necessarily homogeneous throughout the testis and more detailed "insight" into the internal structure of the testis remains desired.

With the aim of obtaining an easy-to-use device giving instant visual evaluation of testicular rigidity, we have developed a prototype electronic system (Electronic Testis Rigidity Tester, ETRT). ETRT measures the pressure of contact with the testis model using a tactile sensor comprised of microsensors that transform pressure values into voltages through alterations of their resistance. Implementation of a software system can graphically depict pressure values over a "floor plan" (color-filled contour plot) of the surface of the model, which comes into contact with the sensor. A color scale shows the magnitude of changes. This pressure map may eventually provide the surgeon with optical information about the rigidity of the testis, reflecting internal structure, and can contribute to a decision about the fate of the testis during operation.

## MATERIALS AND METHODS

### Electronic System

The system comprises five units: the tactile sensor providing the electric signal (mounted on a measuring device), amplification and filtering of the signal, digitalization of the signal, a power supply for the three latter, and a computer program for analysis and visualization (Fig. 1**)**.

#### *Tactile Sensor and Microsensors*

A tactile sensor consists of an array of pressure-sensitive microsensors. Each of the microsensors acts as an electromechanical transducer: the pressure generated from testis model compression changes the resistance of the microsensor (which is supplied by a constant current). Thus, an alteration of voltage difference across the edges of the microsensor is obtained; this is the electric signal that subsequently can be amplified, filtered, digitized, and visualized. The resistance of the microsensors alters in a linear way in response to the force applied perpendicularly on the sensing area[10].

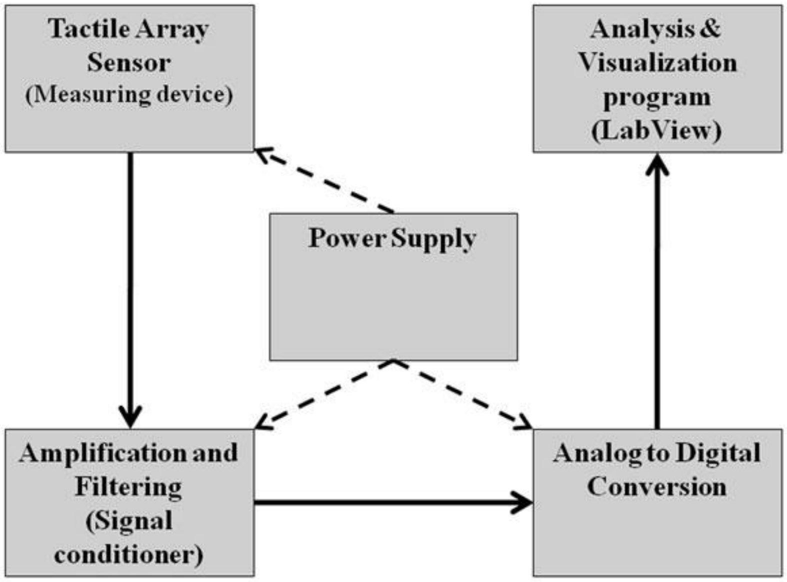

**FIGURE 1.** Block diagram of the proposed electronic system. Data flow is represented with solid arrows between units and power supply with dashed arrows.

Experience with the mechanical TRT that we developed previously[9] guided the choice of microsensors. The TRT measured force resisting a given deformation of the testis ranging between 0 and 133 mNt. From our experiences, we derived an estimation of the measure of the surface area of the testis that would come into contact with the sensor and, of course, we took into consideration cost limitations. Thus, among the sets offered by the manufacturer (Femto Tools, Switzerland), we preferred a sensor that included an array of 3 × 4 microsensors with a pressure range of 0.0068–0.07 kg/cm$^2$ for a sensing area of 20 mm$^2$ for each microsensor. This set corresponded to forces of 13.45–148.46 mNt.

## Measuring Device

We developed a measuring device based on a pincer construction (Fig. 2). The sensor was mounted on the inner surface of one arm (Fig. 2A, a, b); on the inner side of the opposite arm, nothing was mounted. In order to control compression of the testis model, the arms of the pincer were enabled to open and close gradually and precisely by a screw mechanism (Fig. 2A, c). The screw mechanism has marked steps of 1 mm each.

The model or eventually the testis was to be placed between the two arms of the pincer; these approximated each other to come into contact with the testis model. We managed to create a minimum step of compression of 2 mm (vertical to the longitudinal axis of a testis put in the device) by turning the screw mechanism twice. A lesser compression step was technically difficult to achieve.

## Power Supply

Each of the 12 microsensors was supplied by a constant current of 1 mA. To this end, 12 three-terminal, adjustable, bipolar/JFET current sources (LM 134, National Semiconductor, U.S.) were used (Fig. 3).

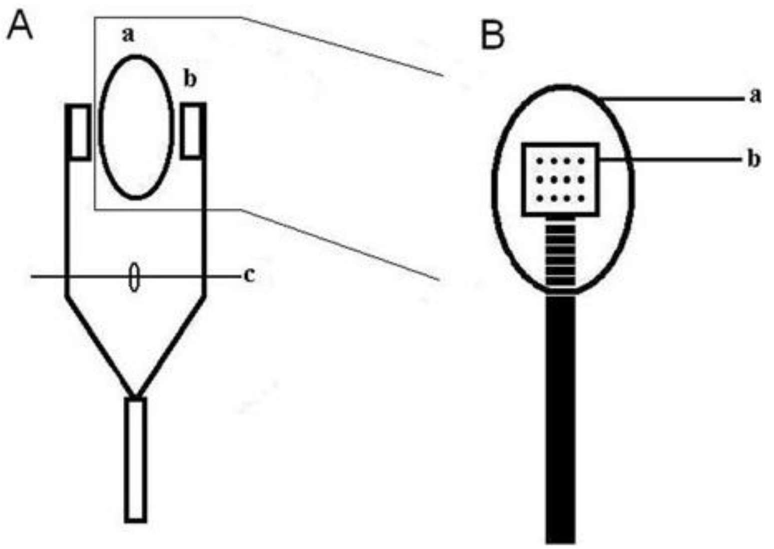

**FIGURE 2.** Graphic depiction of the measuring device of the ETRT. (A) Lateral view of the instrument: the testis model is "captured" by the instrument. (B) Sagittal section of A (magnified and rotated 90° around the vertical axis) showing the microsensors (black dots) that come into contact with the model. a, Testis model; b, tactile sensor; c, shaft controlling the compaction of the model.

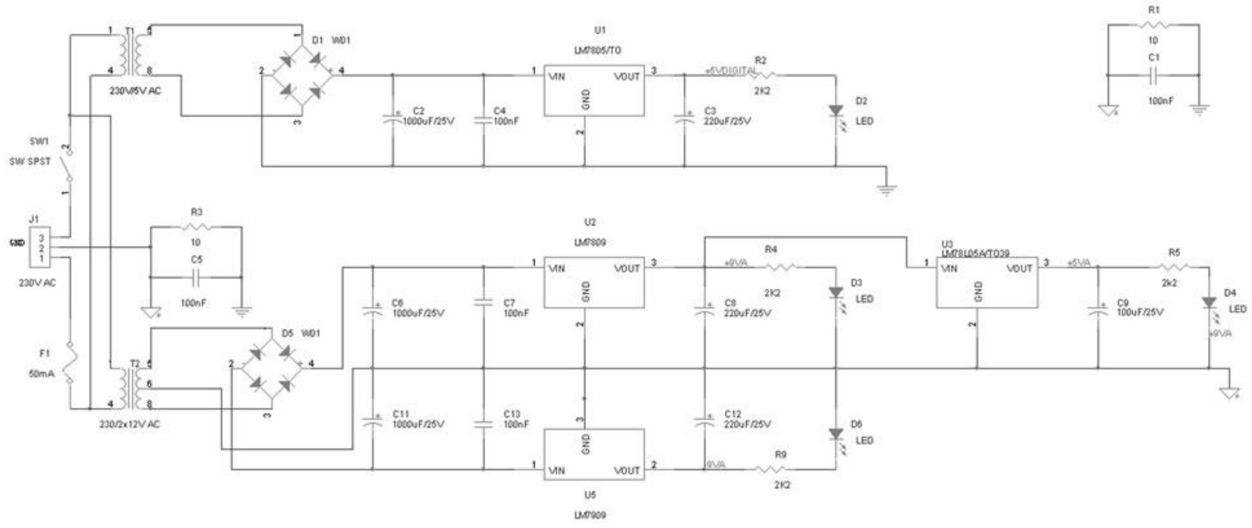

**FIGURE 3.** Diagram of power supply circuit.

The power supply provided symmetrical voltage of 9-V dc for the amplification and filtering unit, 5-V dc for all digital circuits like the Analog to Digital Converter (ADC), and one low-current 5-V dc for the reference voltage of ADC (Fig. 1).

### Amplification and Filtering (Signal Conditioning)

Twelve operational amplifiers (TL071, Texas Instruments, U.S.) enhanced alterations of voltages across each of the 12 microsensors (Fig. 4). The level of amplification was up to 5 V, as imposed by the maximum input voltage of the Analog to Digital Converter. Noninverted amplifiers were preferred because of their high input impedance. Their gain was 100 times as required by the Analog to Digital Converter. The low harmonic distortion and the low noise behavior made the TL071 our choice for this kind of application.

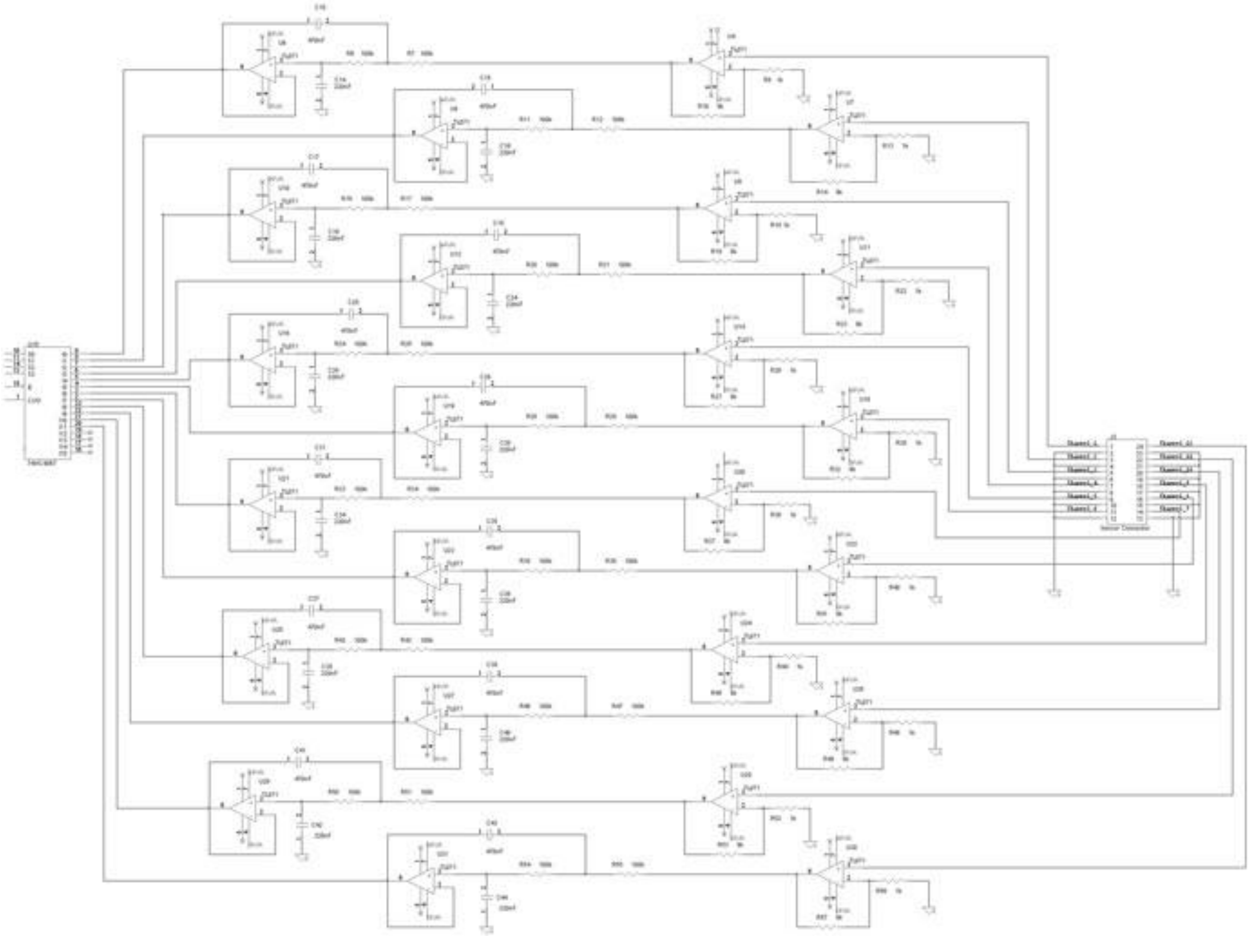

**FIGURE 4.** Diagram of the amplification and filtering circuit**.**

We created a low-pass, second-order (-40 dB/dec), active filter of a cut-off frequency of 4 Hz by adding the appropriate passive components to the TL071 operational amplifiers. The signal before and after amplification and after filtering is shown in Fig. 5. The filtering process guaranteed that any interference signals do not pass the next level of processing, and also acted as antialiasing filters for the next processing unit[18].

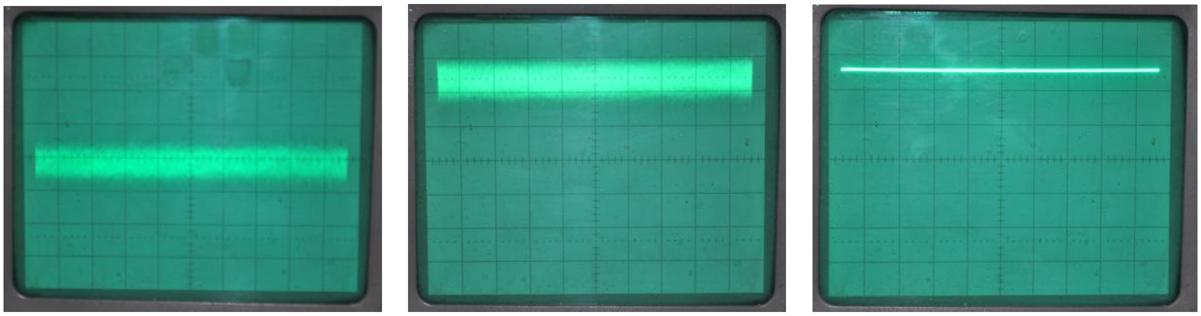

**A** **B** **C**

**FIGURE 5.** Electronic signal output: (A) from the sensor (direct voltage: 250 mV / noise: 10 mV p-p [peak to peak]); (B) after amplification (direct voltage: 2.5 V / noise: 100 mV p-p); (C) after filtering (direct voltage: 2.5 V / noise: <5 mV p-p).

## *Analog to Digital Conversion*

This unit of the electronic system digitized the analog data (amplified voltages). It converted the analog signals into digital signals through the multiplexer (74HC4067, NXP, The Netherlands) and the Analog to Digital Converter (ADC 0800, National Semiconductor, U.S.) (Fig. 6). Then the processor (MEGA 8515, Atmel, U.S.) was used for transmission of the digitized signal via a line driver (MAX 232, Texas Instruments, U.S.) to a personal computer for further processing. The sampling rate was low (10 samples per second) due to the very low frequencies of the original signal.

All units of power supply, amplification and filtering, and analog to digital conversion were assembled on a printed circuit board (data acquisition card, DAQ).

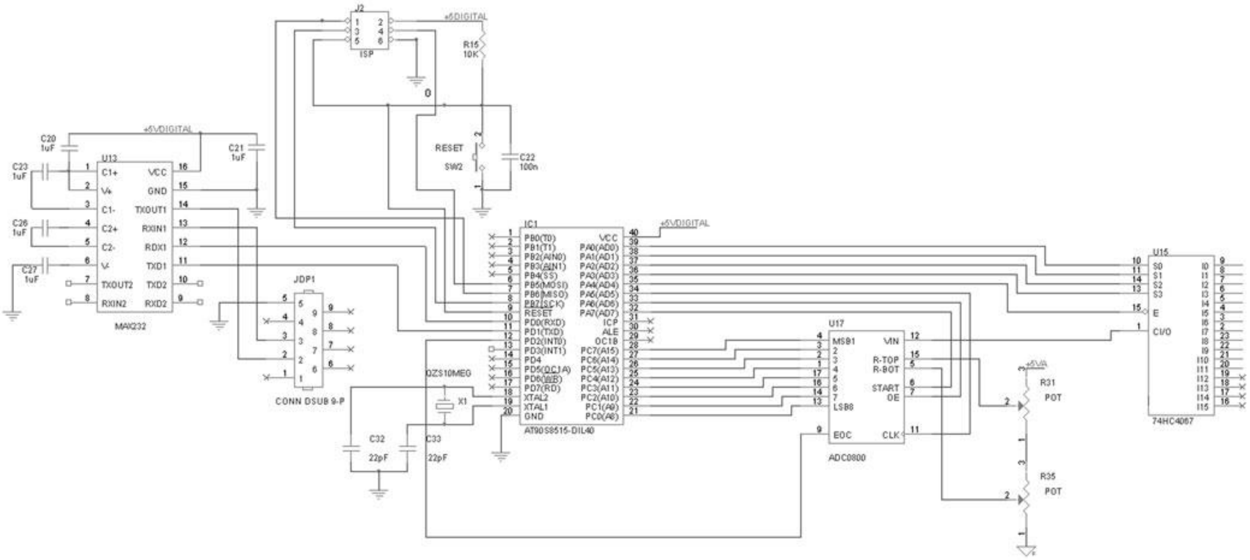

**FIGURE 6.** Analog to digital conversion circuit**.**

### Computer Analysis and Visualization

A computer accepted the digitized data from the DAQ card, processed them, and presented them in graphic format for evaluation of results. The sampling rate of the Analog to Digital Converter (10 samples per second) generated a time vector from the data obtained by each microsensor. The 12 vectors (one from each microsensor) created a 12-row table of measures; this table was continuously updated (10 times per second).

These measures were constantly transferred from the DAQ card to the computer by the series protocol RS232. The computer processed the received data by applying a high-level language, such as LabView (Laboratory Virtual Instrument Engineering Workbench, version 8, National Instruments, U.S.)[11]. The program handled the series of data (time vectors) to create a two-dimensional contour plot of the sensing area of the microsensors.

The graphic output was given as a colorful orthogonal figure representing the cross-section of the surface of the testis model coming into contact with the sensor. The system was calibrated so that each pressure value was coded by a different color. Warmer colors in the contour plot represented higher pressure values and cooler colors represented lower pressure values.

## Phantom Construction and Testing

### Phantom Construction

As testis models, we used a set of four balls of 40-mm diameter, volume 33.5 $cm^3$, and relative density 0.84 $g/cm^3$. Using a minidrill, a hole of 2-mm diameter was drilled into each model up to its center (Fig. 7A). Then, with the milling accessory of the minidrill, material from the center of the ball was removed to empty a space of about 10 $cm^3$ (24-mm inner diameter). Using a specific volume of sand, this space was verified to be the same for all four balls.

A different quantity by weight of acrylic glue was injected into each phantom ball (5, 10, 15, and 20 g) (Fig. 7B**)**. This glue, in the form of foam, of relative density of 1.9 $g/cm^3$, filled the empty volume inside the ball and solidified 24 h later (Fig. 7C). The greater the quantity of glue injected into the ball, the higher the density of the foam, resulting in greater rigidity after the solidification of the acrylic glue.

We also used a model of the shape of a prolate spheroid, with the polar axis longer than the equatorial diameter (i.e., like a rugby ball), with the following physical characteristics: dimensions 40 × 50 mm, volume 48 $cm^3$, weight 37 g, relative density 0.77 g/ $cm^3$. An empty space of 10 $cm^3$ (inner diameter 24 mm) was drilled into the center of this phantom. This space was injected with 7 g of the same acrylic glue. The prolate spheroid model was to be used apart from the set of the four balls mentioned above.

### Testing of Rigidity by an Independent Method

#### Mechanical Method

For the independent testing of the phantom ball rigidity, a mechanical method was used measuring the force needed for a known constant deformation. An electric weight scale (Delonghi, Italy) was used as a dynamometer, fixed on one arm of a large micrometer (Rabone Chesterman, U.K.). The scale and micrometer were tilted horizontally and placed on a support so that the weight of the ball did not affect measurement on the scale. The distance between the measuring surface of the scale and the movable end of the micrometer bar was set to 40 mm. A ball was then set, without being compressed, between the measuring surface of the scale and the movable end of the micrometer (Fig. 8A). Care was also taken to place the ball with the axis of the hole from the drilling procedure vertical to the axis of the micrometer bar, in order to avoid biasing the measurements that could possibly arise from the orientation of the area of the hole. Then, by turning the calibrated screw mechanism, a displacement of 2 mm was applied to the

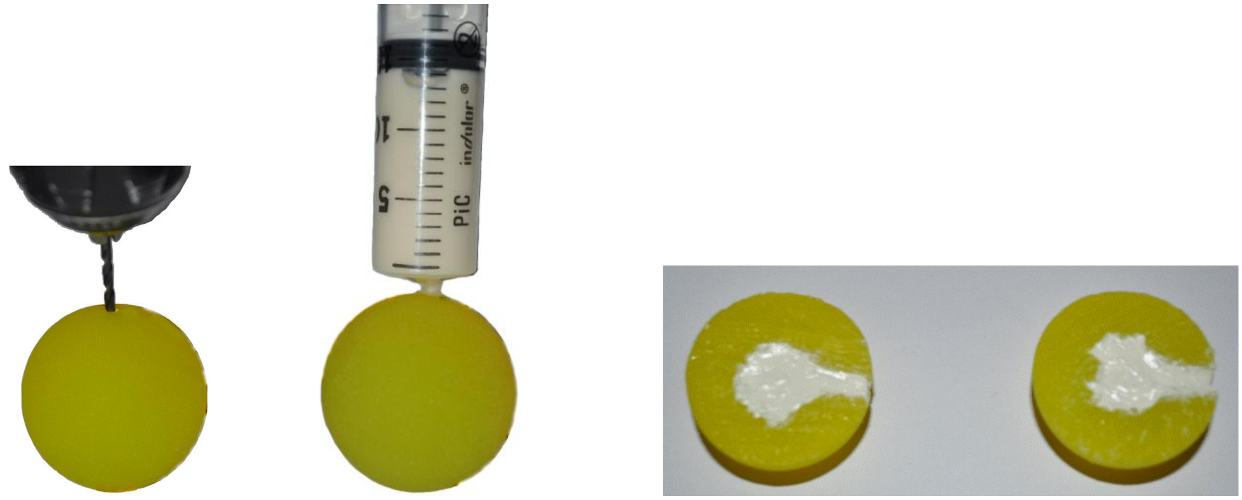

A B C

**FIGURE 7.** Construction of phantoms. (A) Each ball was drilled and a volume of 10 $cm^3$ was emptied. (B) A different quantity of acrylic glue was injected into each ball. (C) Section of phantom along the axis of drilling after glue consolidation. The foam of the glue filled in all empty spaces; 5 g of glue was injected into the left ball, 20 g into the right ball.

movable end of the micrometer, compressing the ball. The electric weight scale was used to quantify the force needed to achieve this constant known deformation for each ball, as the measurement in grams is proportional to the force applied on the ball.

### Tactile Single Sensor

An internal test was also performed to verify that the electronic pressure sensor was able to quantify the differing rigidities of the four phantom balls in the same manner as the mechanical method (measuring force for a known deformation). For this goal, a single sensor (Tecksan, U.S.) was selected with a sensitivity of milliNewtons, similar to that of the microsensors of the tactile sensor used in the ETRT system. The single sensor was connected to a digital multimeter, able to show force measurement in pounds (lbf). The electronic system described earlier was not used; thus, only the function of the single sensor was tested here.

Each phantom ball was inserted between the measuring arms of a micrometer that was initially opened to 40 mm so that the ball was not compressed. Again, the axis of drilling was set vertical to the axis of the micrometer. Then, the same constant deformation of 2 mm was applied by turning the screw mechanism of the micrometer. The force needed to reach the constant known deformation was quantified this time by the sensor placed at the position where the fixed end of the micrometer came into contact with the phantom ball, as shown in Fig. 8B.

## *Testing Protocol with Tactile Microsensors*

Here the entire system of ETRT was used — the measuring pincer device with a sensor comprised of an array of 12 microsensors — and was connected to the electronic system described above. First, the prolate spheroid phantom was tested to examine whether the whole ETRT system would be able to detect the central, more rigid region and display it in the graphic layout. Then, the four phantom balls were measured to

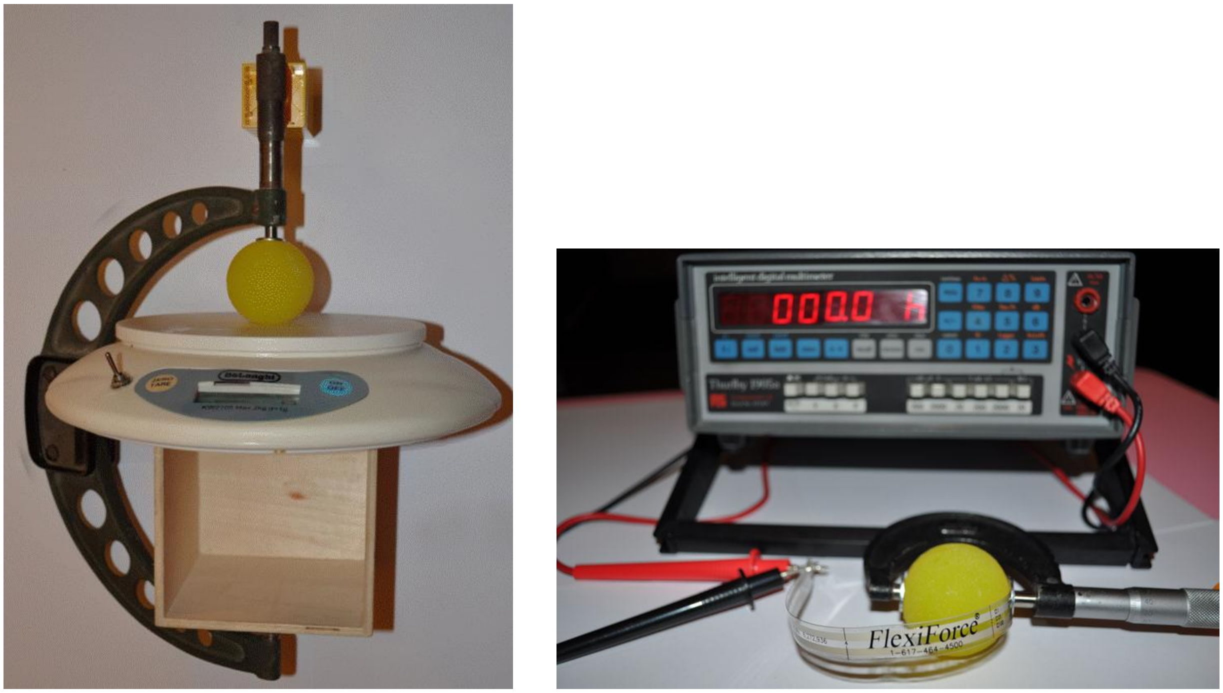

**A** **B**

**FIGURE 8.** Testing of phantoms' rigidity. (A) Mechanical method, using a large micrometer for 2 mm and an electric weight scale as dymanometer, set horizontally to avoid the weight of the ball. (B) Testing with a single sensor of similar sensitivity with microsensors used on the pincer of ETRT, connected to a multimeter (the electronic system of ETRT not used).

investigate whether the ETRT was able to reveal and display a different pressure layout for the four balls of known different rigidity in their central areas.

Before each measurement, the two inner surfaces of the pincer were brought to a distance of 40 mm by the screw mechanism. The phantom was inserted, without being deformed, between the inner surfaces of the pincer coming into contact with the sensor on one side and with the pincer plate on the other. The prolate spheroid phantom was placed with the long axis parallel to the legs of the pincer. The axis of the drilling that created the hole (see above) was set parallel to the plates of the pincer. The distance between the plates was then reduced by 2 mm, compressing the phantom. The applied force on the phantom and the pressure on the contact surface varied depending on the rigidity of the measured item. When the rigidity was greater, a greater force was required to reach the constant known deformation. The sensor bearing the microsensors measured the pressure applied on the area of the surface of the phantom that was in contact with the pincer, quantifying the force needed for the constant deformation and, thus, the rigidity of the phantom.

## RESULTS

The four phantom balls that were mimicking testes, injected with different quantities of foam glue, were all measured by using a micrometer and an electric weight scale for the force needed to reach a 2-mm deformation, as described above. Results are shown in Table 1 (mechanical method). As can be seen, greater quantities of injected glue corresponded to a greater force needed to achieve the known deformation, thus quantifying the rigidity of the phantom ball. Therefore, the claim of increasing rigidity according to the injected quantity of glue was verified.

**TABLE 1**
**Testing Rigidity of Phantom Balls Injected with Glue using a Mechanical Method (Independent Method), and a Single Sensor and Multimeter (Test of Tactile Sensor)**

| Ball Number | Quantity of Glue (g) | Mechanical Method (Electric Weight Scale and Micrometer) | | Single Sensor Connected to Multimeter (ETRT System Not Used) | |
|---|---|---|---|---|---|
| | | Measured Weight (g) | Corresponding Force (mNt) | Measured Weight (lbf*= g) | Corresponding Force (mNt) |
| 1 | 5 | 22 | 215 | 0.050 = 23 | 226 |
| 2 | 10 | 26 | 254 | 0.059 = 27 | 265 |
| 3 | 15 | 32 | 314 | 0.075 = 34 | 333 |
| 4 | 20 | 38 | 373 | 0.088 = 40 | 392 |

* The output of the multimeter was in pounds (libres); conversion in grams follows.

The four balls were tested with a single sensor, as described above, in order to examine whether a tactile sensor of proper sensitivity, not connected to the electronic system, but only to a multimeter, would give similar force measurements for a 2-mm deformation. From the results shown in Table 1 (single sensor connected to multimeter), it is evident that this sensor gave force measurements very close to those obtained by the mechanical method of altered rigidity detection.

The ETRT system was perfectly capable of producing a graphic layout of a pressure map of the models tested. Results of rigidity measurements over the tangential cross-section of the surface of the phantom coming into contact with the sensor are shown in color scale (Figs. 9 and 10). We calibrated the pressure scale using LabView; the gray color represented the minimum value of 0.0068 kg/cm$^2$ and the red color represented the maximum value of 0.07 kg/cm$^2$.

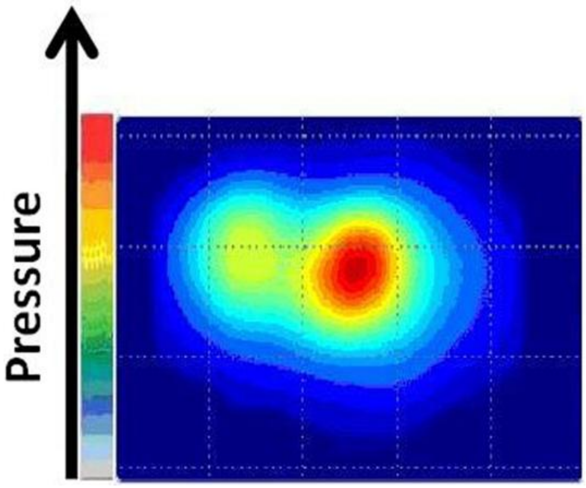

**FIGURE 9.** Graphic output of pressure measured as a response to predetermined deformation in a testis model. Colors of testicular areas on the floor plan of the model represent pressure values. The color scale on the left shows the correspondence of colors and pressure. The gray represents the minimum value of 0.0068 kg/cm$^2$ and the red color represents the maximum value of 0.07 kg/cm$^2$. Warmer colors represent areas of high pressure, while cooler colors represent areas of lower pressure. Pressure values reflect the rigidity of the phantom in the respective areas of the plan.

Fig. 9 shows the contour plot of the pressure measurements obtained from the testis model of a prolate spheroid shape. The centrally located black area had pressure values exceeding the maximum value (0.07 kg/cm$^2$). The area marked with dark red color represented the sites of maximum pressure; areas of lesser pressure are shown with cooler colors, e.g., light blue. These color-coded pressure values indicate areas of differing rigidity within the testis model.

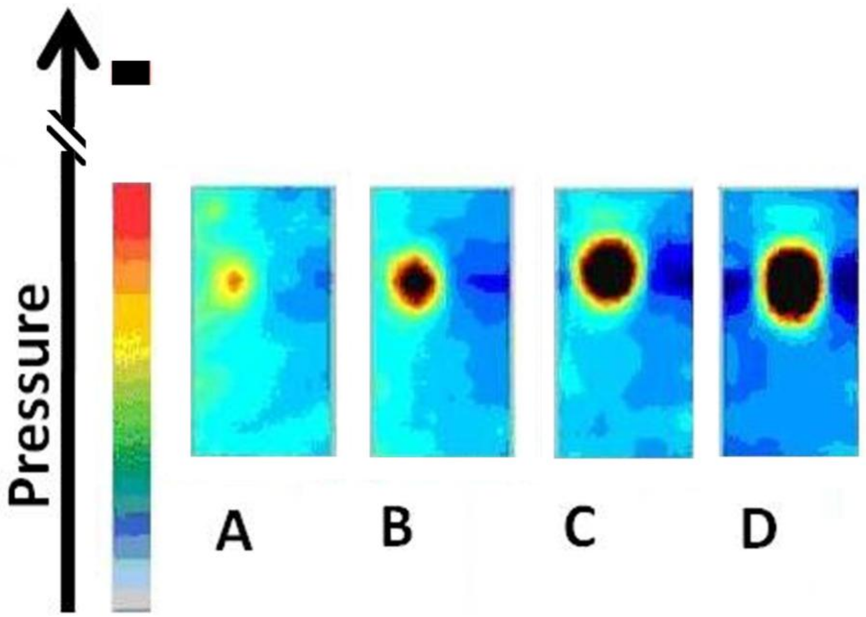

**FIGURE 10.** Comparative view of four graphic outputs (A–D) from testicular models with increasing rigidity. The color scale on the left shows the corresponding pressure measured as a reaction to predetermined deformation of the model.

The color-filled contour plots obtained from the other four testis models (spherical shape) are shown in Fig. 10. A different color pattern can be seen in comparison with Fig. 9. In Fig. 10, this pattern consists of differing distributions of colors, dependent on the consistency of each model. The testis model with the most rigid core (more glue within the model) is shown with a wider central black area (Fig. 10D). The model with the most rigid central area had a wider area in contact with the sensor because the surface bearing the microsensors was flexible and was deformed when in contact with the more rigid models.

The surrounding blue areas are sections of the sensor that do not come into contact with the testis. Among each of the four models presented in Fig. 10, we can observe differences in the color tone of the surrounding areas (outside the surface of the model that comes into contact with the sensor). As can be seen, the model with the hardest core exhibited a deeper blue with less of a light blue surrounding area, in comparison with the other three models; this indicates a slightly increased pressure in the surrounding area. We attribute these differences to the (plastic) membrane cover of the microsensors. Apparently, when high-pressure areas were measured, a pressure distortion over the membrane was produced in neighboring areas.

The ETRT system managed to detect the differences in rigidity between each of the four testis models. An observer performed a blind palpation evaluation of the rigidity of the four models. He was able to detect differences between testis models D-C, D-B, D-A, C-B, and C-A, but was unable to detect any difference in rigidity between models B and A.

## DISCUSSION

We aimed to provide a device for intraoperative evaluation of testicular elasticity for intraoperative use in cryptorchidism and torsion. Histological lesions alter testicular internal structure, which is reflected in rigidity of the testis**.** Phantoms resembling the testis, of known different rigidity, were compressed in a predetermined manner. A tactile sensor measured the pressure applied on the surface of the phantom, quantifying the force needed for the deformation and, thus, the phantom rigidity. The sensor transformed pressure values into alterations of its resistance and, with the use of an electronic circuit, the system was able to display the direct optical output of a pressure map of the phantom.

The proposed electronic system (ETRT) has several advantages. It is a simple and inexpensive device for detection of alterations of elasticity, mimicking the physical distortion caused by physical examination. Graphic depiction of pressure values over a map filled with colors is a user-friendly application. The manufacturing characteristics of the applied tactile sensor provided three times higher sensitivity than the human stereognostic sense[10]. Thus, ETRT would be able to detect differences in testicular rigidity undetectable with palpation, thus helping the surgeon to make decisions about surgical management that can be based on objective data. The system, except for the instant view of data, is able (due to the LabView program) to provide the user with a graphic environment containing tools for measurement collection, individual electronic instruments control, data presentation, and analysis. The interactive environment allows experimentation on data and offers a convenient means of error detection[11].

We have had several issues to consider during this initial development of ETRT. One question has been whether the edge measuring device of ETRT is of appropriate size for future testing in testes of rats and humans. The diameter (40 mm) of the phantoms tested here exceeds the "horizontal" diameter (vertical on longitudinal axis) of adult human testes and, undoubtedly, that of children and rats. The measuring device was created with the aim that the whole system would, at a later time, first be used experimentally in rat testes and finally be tested clinically in pediatric operations (we note here that the size of the testis of young children is not much different from those of adult rats). All of these different size testes can be measured by the device because the surface of the testis that comes into contact with the sensor, represented as the cross-section in the colorful orthogonal figure of the output, is a very small proportion of the whole surface of the testis.

The selection of microsensors of proper sensitivity was based on measurements obtained by the mechanical TRT that we previously developed[9] and is therefore justified. The density of microsensors on the sensor board was in part constrained by cost factors. These microsensors produced good preliminary results; however, the maximum of their sensitivity was easily surpassed during the testing protocol in phantoms of increasing rigidity (black color in Fig. 10B, C, D). The single sensor tested with the multimeter alone was of similar sensitivity with the chosen microsensors, and gave force measurements very close to those obtained by the weight scale and micrometer (mechanical method).

Construction of the current phantoms of different rigidity can be judged to be rather unrefined. However, the design of this study included a series of progressive tests at various levels, each testing a step in the sequence of ETRT development. Injection of foam glue was successful in creating a center volume of altered rigidity, as shown by the independent method of mechanical quantification (using a micrometer and an electric weight scale). Testing the phantoms at first with a single sensor (of sensitivity similar to that of microsensors used afterwards) connected to a multimeter and without using the complete ETRT system, assured the function of the tactile sensor in the framework of quantification of pressure alterations. Use of the sensor with a $3 \times 4$ array of microsensors (of proper sensitivity, known by development of a similar mechanical device[9]) connected to the electronic ETRT system elaborated here, succeeded in showing the altered rigidity in a large phantom mimicking the human testis (protate spheroid shape). Finally, the ETRT system accomplished the aim of discriminating between the four phantom balls of proven different elasticity and exceeded the discrimination of an examiner's palpation.

In these tests, care was taken to avoid having the axis of the drilling process vertical to the surface of measurement so that any bias of this "damaged" area would be avoided. Alterations in the surrounding blue and green color scale areas representing the surface of the sensor not coming into contact with the phantoms is a minor problem, which will be repaired in further development, so that the resulting contours of ETRT graphic layouts do not differ between testes of different rigidity.

That ETRT is safe for future use on testes remains to be proven. We assume that a 2-mm compression will not produce any harm, but this, of course, remains to be proven by (an) experimental study examining testicular histology. However, our experience in the operating room with human testis

(children and adolescents) and in the laboratory with rats testes tested with the mechanical TRT developed previously[9] provides some evidence that this degree of compression most likely will not be harmful.

Several researchers have already developed devices based on tactile imaging for evaluating other solid organs (liver[12], breast[13,14], prostate[15], vaginal wall[16], etc.); these have been evaluated in clinical studies with promising results. More sophisticated systems of elasticity imaging, using air jet[3], water jet[17], or ultrasound vibration[18], for tissue disturbance and measured by MRI[2], ultrasound[1,17], or optometrics[3], were beyond our aim to create a low-cost, flexible electronic system with a single probe for use in an intraoperative setting. Other systems that have been developed for intraoperative use, such as a suction system for mechanical imaging of the human liver[12], could not be applied to our setting due to the small size and concavity of the testis.

The proposed electronic system could be applied to surgical specialties involved in the treatment of testicular diseases, such as pediatric surgery and urology, after, of course, being tested in experimental animal studies. Our intention is to develop an electronic instrument with even more sensitive tactile sensors for intraoperative use in humans. With the incorporation of artificial intelligence techniques and the association of rigidity values to histologic data, this instrument could automate and standardize the measurement process. After appropriate studies, it could also be used to predict testicular functional status and future viability of the testis.

## ACKNOWLEDGMENTS

Dr. Elissaios Kontis substantially helped us with the design and experiments, and with photographs in the current manuscript. The authors are also grateful to Carol Froman for editorial assistance and to Konstantinos Myrillas, MEng, for correcting this manuscript from an engineering point of view.
